# Posterolateral Endoscopic Lumbar Decompression Rotate-to-Retract Technique for Foraminal Disc Herniation: A Technical Report

**DOI:** 10.1155/2019/5758671

**Published:** 2019-02-17

**Authors:** Eun Sang Soo, Chachan Sourabh, Lee Sang Ho

**Affiliations:** ^1^Department of Orthopaedics, Wooridul Spine Hospital, Gangnam-gu, Seoul, Republic of Korea; ^2^Department of Neurosurgery, Wooridul Spine Hospital, Gangnum-gu, Seoul, Republic of Korea

## Abstract

**Background and Study Aim:**

Foraminal disc herniations present the unique surgical challenge for exiting nerve root retraction and decompression. The aim of current study is to describe an innovative maneuver and evaluate its usefulness for endoscopic decompression of foraminal disc herniations.

**Material and Methods:**

A retrospective review was performed including cases of foraminal disc herniations who underwent endoscopic discectomy utilizing the rotate-to-retract technique. Data on patient demographics and improvement in VAS/ODI scores were collected and analyzed statistically.

**Results:**

There were ten patients (three male; seven female) in the final analysis. Seven procedures were done at the L4-L5 level, two were done at the L5-S1 level, and one was done at the L3-L4 level. The average VAS scores improved from preoperatively 7.5 to postoperatively 4.4 (p= 0.001). The mean preoperative ODI was 67.8 and improved to 26.6 postoperatively (p< 0.001). None of the cases reported any neurological or dural complication.

**Conclusion:**

Foraminal disc herniations can be safely and adequately addressed endoscopically with the use of rotate-to-retract technique.

## 1. Introduction

Posterolateral endoscopic lumbar decompression (PLELD) is fast becoming the procedure of choice for surgical management of lumbar disc herniations [1–7]. Endoscopic discectomy techniques have produced surgical results similar to those of other discectomy techniques, while offering various advantages like avoidance of general anesthesia, preservation of paravertebral soft-tissues, faster rehabilitation, and better clinical results overall [1–7]. Cases of foraminal disc herniation (FDH) present the unique surgical challenge for exiting nerve root retraction and decompression [8–11]. Irrespective of the surgical technique used, the clinical outcome can be significantly affected by both technique of exiting nerve visualization/retraction and adequacy of decompression [8–11]. Use of appropriate exiting nerve retraction and visualization technique is paramount to adequate decompression [8–11]. The aim of this paper is to report an innovative maneuver, the “rotate-to-retract technique,” for safe retraction and decompression of the exiting neural structures during PLELD in cases of FDH.

## 2. Material and Methods

This study is a retrospective review of prospectively collected data extracted from local spine registry records. All surgeries were performed between February 2015 and October 2017 by a single spine surgeon (SSE). Inclusion criteria included all patients who were diagnosed with lumbar radiculopathy due to foraminal disc herniations, failed conservative therapy, and underwent PLELD. Exclusion criteria were revision cases, patients with multilevel radiculopathies/disc pathologies and calcified herniated discs. Data on patient demographics and level/side/duration of surgery were recorded. Clinical outcomes were evaluated using VAS/ODI scores collected preoperatively, postoperatively, and at final follow-up.

### 2.1. Surgical Technique

**Figure 1:**
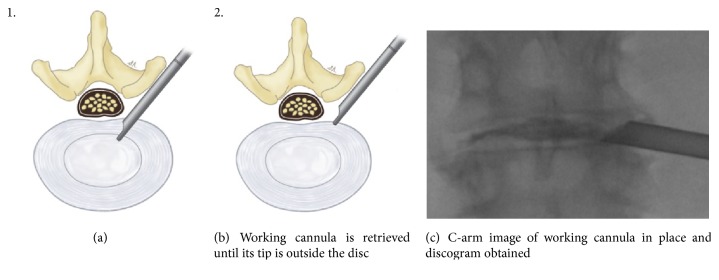


**Figure 2:**
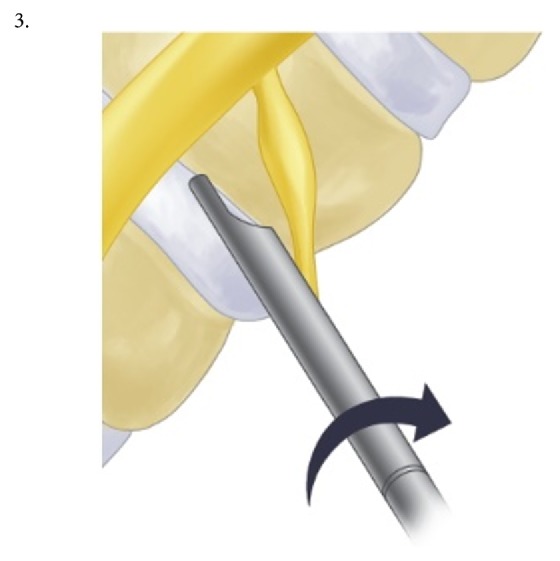
Working cannula is rotated such that tip and opening of bevel are on the cranial side.

**Figure 3:**
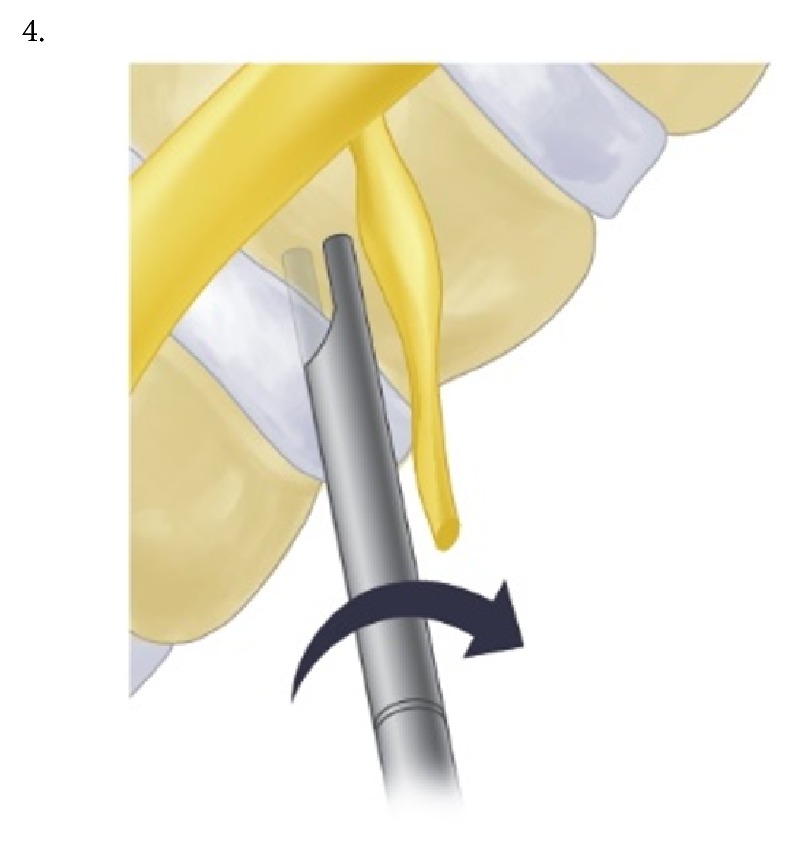
Working cannula is rotated clockwise.

**Figure 4:**
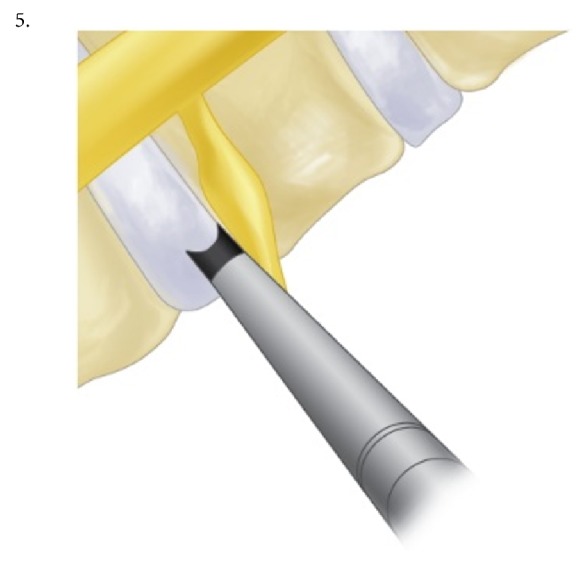
Working channel is placed in most lateral part of Kambin's triangle and bevel is retracting the exiting root.

**Figure 5:**
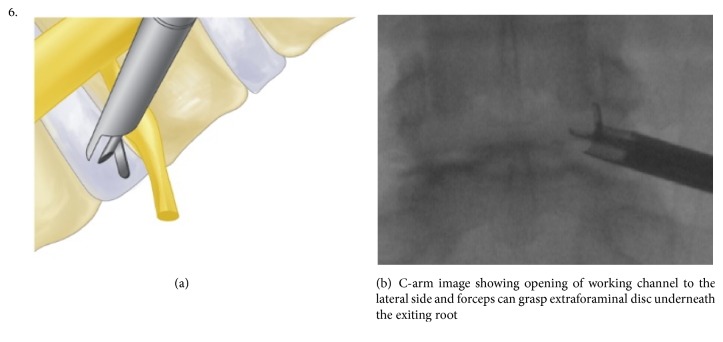


**Figure 6:**
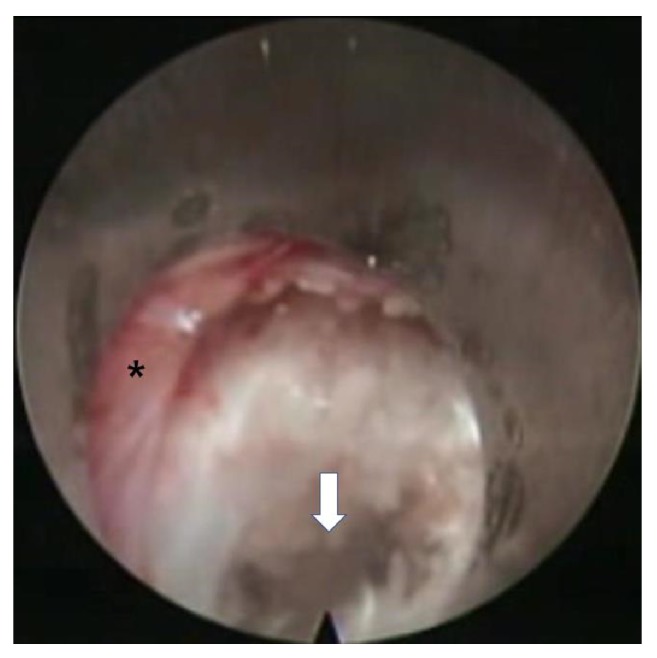
Endoscopic image showing exiting nerve root (*∗*) and disc space (arrow) after decompression.

The procedure was performed under local anesthesia with mild sedation. The patient was positioned prone. A standard lumbar endoscopic instrument set (TESSYS®, Joimax®, Hamburg, Germany) was used. The surgical steps were as follows:

1. The skin entry point and trajectory of the endoscope were planned based on the axial magnetic resonance (MR) images. The surgeon preferred to use a more direct trajectory towards the herniation resulting in the skin entry point about 7-8 cm from the midline with a steep angle of approach.
2. The path of the endoscope was infiltrated with local anesthesia.
3. An 18 G spinal needle was inserted under fluoroscopy guidance along the preplanned trajectory and needle tip is positioned in the spinal canal.
4. Epidurography was performed to confirm the location of the neural structures.
5. After confirmation of correct needle tip position, a guide wire was introduced via the spinal needle, followed by an obturator and a beveled working cannula (Figure 1(a)).
6. The whole procedure was performed under fluoroscopy guidance. After satisfactory positioning of the working channel, a 25° endoscope was introduced.
7. To safely approach the foraminal disc, rotate-to-retract technique was employed:
  - The working cannula was retrieved until its tip was outside the disc (Figures 1(b) and 1(c)).
  - The working cannula was rotated such that the tip and opening of the bevel were on the cranial side (Figure 2).
  - It was then rotated clockwise, which resulted in spontaneous retraction of the exiting nerve root (Figure 3).
  - The working channel was placed in the most lateral part of Kambin's triangle with its bevel tip retracting the exiting root (Figure 4).
  - By rotating the opening of the working channel to the lateral side, endoscopic forceps could be used to grasp the extra-foraminal disc herniation underneath the exiting root (Figures 5(a) and 5(b)).
8. Rest of the discectomy was performed and concluded in the standard manner.
9. Intraoperatively, exiting nerve root decompression could be assessed by direct inspection with endoscope (Figure 6).

### 2.2. Statistical Analysis

Pre- and postoperative VAS and ODI scores were calculated and statistically compared using paired t-tests. P value <0.05 was considered statistically significant. All analyses were performed using SPSS (IBM SPSS Statistics for Windows, Version 22.0. Armonk, NY: IBM Corp.).

## 3. Results

**Figure 7:**
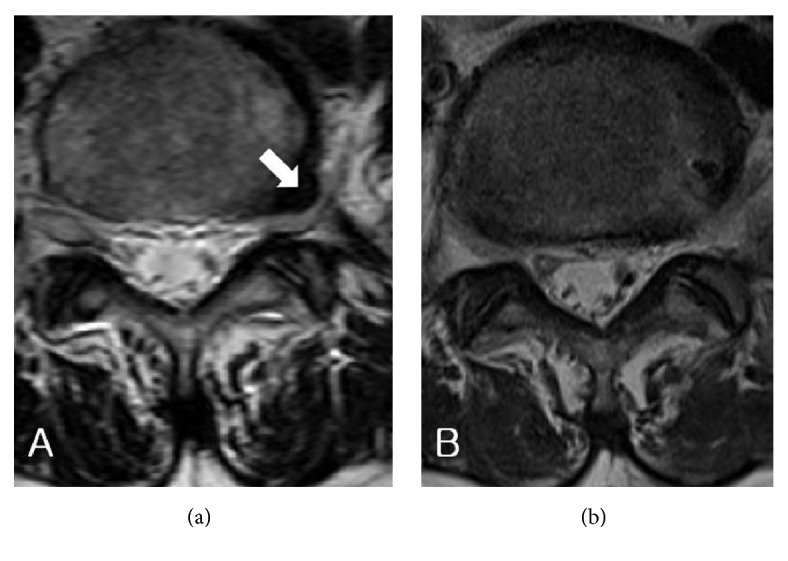
(a) Pre-op axial MRI showing left-side extra-foraminal disc herniation (white arrow) at L5-S1 level. (b) Post-op axial MRI showing removal of extra-foraminal disc herniation.

There were ten patients (three male; seven female) in the final analysis, with an average age of 62.4 years (range 44-80 years) [Table 1]. The average duration of surgery was 52.5 minutes (range 35-70 minutes). Of the ten cases, six were operated on the right side and rest on the left side. Seven procedures were done at the L4-L5 level, two were done at the L5-S1 level, and one was done at the L3-L4 level. The mean follow-up period was 5.6 months (range 4-8 months). The average VAS scores changed from preoperatively 7.5 (range 6-8) to postoperatively 4.4 (range 2-8). The change in VAS scores was statistically significant (p= 0.001). The mean preoperative ODI was 67.8 (range 42–84) and improved to 26.6 (range 16-55) postoperatively, which was statistically significant (p< 0.001) [Table 2]. All the patients underwent an immediate postoperative MRI, which showed successful removal of the herniated disc fragment and good decompression of the exiting nerve root in all the cases (Figure 7). None of the cases reported any neurological or dural complication. All the cases showed good improvement in ODI scores. All except one case reported good postoperative improvement in pain scores.

## 4. Discussion

In the current series, use of rotate-to-retract technique during PLELD resulted in complete removal of the FDH. This technique offered effective and safe retraction of the exiting nerve root in the Kambin's triangle [12]. The authors have reported use of beveled working cannula to effectively remove the inferiorly migrated disc herniation using transforaminal approach [13]. With all the steps of rotate-to-retract technique, surgeon can address a variety of disc lesions: canalicular, foraminal, axillary (exiting root), upmigrated, and extra-foraminal (underneath the medial border of exiting nerve root).

Compared with central disc herniations, foraminal disc herniation discectomies (microscopic/endoscopic) have a reportedly higher postoperative incidence of remnant radicular pain and paresthesia [14]. The authors postulate that the inferior outcomes of FDH discectomies can be attributed to DRG (dorsal root ganglion) manipulation. Furthermore, removal of FDH can result in disc height decrement, segmental instability, and foraminal stenosis [7, 11, 12].

The term foraminal disc herniation (FDH) is interchangeably used with far lateral, extra-foraminal, and extreme lateral disc herniations [8–11]. Since initial reporting of its clinical manifestations by Abdullah et al., both detection and treatment rates of FDH have increased consistently [11]. FDH is both a diagnostic dilemma and a surgical challenge [8–11]. The diagnosis is complicated by ambiguous clinical features mimicking a posterolateral disc at the level above [8–11]. Furthermore, as multilevel disc herniation is not uncommon, missing a foraminal nerve root compression is easy [8–11]. This also explains highly variable reported incidence of FDH (0.7-11% of all lumbar disc herniations) [8–11]. The advent of MRI has significantly increased FDH detection and successful surgical treatment rates [8–11].

The surgical management of FDH is challenging due to an anatomically constrained area with associated higher risk of neural injury and iatrogenic segment instability [8–11]. All of these features combined produce high chances of failed back surgery in cases of FDH [8–11]. Various modifications of standard open and microsurgical techniques have been described for the management of FDH [8–11, 15–18]. The use of conventional midline open surgery approaches for FDH, although familiar and comfortable for surgeons, is surgically counterintuitive, requiring removal/exposure of central/paracentral structures for removing pathological material which is mainly extra-canalicular [8–11, 15–18]. Although paraspinal open surgical approaches make more sense surgically, they are unfamiliar to many surgeons and also pose a risk of iatrogenic instability of facet joints [8–11, 15–18]. Open transforaminal approaches give good exposure too but are more invasive, result in iatrogenic instability, and are associated with higher morbidity [11, 12]. Combined intra- and extra-canal open surgical approaches have also been described but are discouraged due to excessive soft-tissue dissection/retraction and longer operative times [8–11, 15–18]. Midline contralateral approaches have also been described to achieve good decompression of FDH but are associated with compression of neural structures [19].

Several studies have reported successful outcomes with endoscopic removal of FDH [8–11, 16]. PLELD offers the advantage of minimal soft-tissue disruption, no bone resection, less bleeding, low chances of iatrogenic instability, shorter operation times, and faster rehabilitation, but are limited by a smaller field of vision and constrained anatomy which significantly increases the risk of exiting nerve root injury and inadequate decompression [8–11, 16]. Various modifications and maneuvers have been described to overcome the specific surgical challenges associated with endoscopic removal of FDH [8–11, 16]. The use of a standard method to retract nerve roots safely and securely away from the operating field will help in minimizing the complications. Furthermore, a standardized and adequate nerve retraction technique may result in faster herniotomy and decrease in overall surgical time.

The above described rotate-to-retract technique is a simple-easy-to-learn maneuver involving the use of beveled end of the working cannula to safely retract the exiting nerve root in its axilla, permitting complete removal of the pathological disc material. Use of the above-mentioned technique has resulted in good surgical outcomes in the current study. However, the small number of cases analyzed and lack of comparison with other techniques may limit the utility of the current study. Further studies including a larger number of cases can help in identifying the role of various other factors like disc height, superior articular process encroachment, bony spur on the lower end plate of cranial vertebrae, and concomitant lateral recess stenosis. The authors would also like to point out that this technique is probably being used by many spine endoscopists but has never been formally described in literature. The authors believe that a standardized description of this useful technique would be helpful in teaching safer methods of endoscopic spine surgery to beginners.

## Figures and Tables

**Table 1:** Demographic and operative data.

Sr No.	Gender	Age	level	side	op time (min)
1	f	80	45	Right	50

2	f	62	56	Left	55

3	f	64	45	Right	60

4	m	75	34	Left	45

5	m	44	45	Right	70

6	f	66	45	Right	50

7	f	68	45	Right	45

8	f	47	45	Right	35

9	f	45	45	Right	55

10	m	73	56	Right	60

**Table 2:** Clinical outcome data.

Sr No.	preop-VAS	preop ODI	postop-VAS	postop-ODI	f/u (month)
1	8	82	3	20	6

2	7	75	3	18	6

3	8	80	8	46	6

4	8	82	5	55	6

5	6	42	4	24	6

6	7	54	2	33	4

7	8	73	5	16	4

8	7	53	3	20	4

9	8	53	8	18	8

10	8	84	3	16	6

## Data Availability

The data used to support the findings of this study are included within the article in the form of table (Table 1).

## Supplementary Materials

Supplementary Materials Intraoperative endoscopy video showing rotate-to-retract technique being used during posterolateral endoscopy discectomy.
